# Sex-dependent protective responses of taurine in ameliorating radiation-induced intestinal injury: gut microbiota reprogramming in males and estrogen-dependent immunity in females

**DOI:** 10.7150/ijbs.137118

**Published:** 2026-08-24

**Authors:** Xudong Ding, Jia Du, Zhaoyu Wang, Lu Lu, Saijun Fan

**Affiliations:** Institute of Radiation Medicine, Chinese Academy of Medical Sciences and Peking Union Medical College, Tianjin Key Laboratory of Radiation Medicine and Molecular Nuclear Medicine, Tianjin 300192, China.

**Keywords:** taurine, ionizing radiation, intestinal injury, gut microbiota, AICDA, sexual dimorphism

## Abstract

Radiation-induced intestinal injury (RIII) limits abdominal and pelvic radiotherapy. Safe and effective radioprotectors that do not protect tumors remain an unmet need. Here, we examined the radioprotective effect of taurine (Tau) in mice subjected to whole-abdominal irradiation (WAI) and whether this effect depends on sex. Tau treatment improved post-irradiation survival, preserved intestinal stem cells, prevented mucosal barrier breakdown, and reduced systemic inflammation. These effects, however, were sex-dependent. In male mice, Tau altered the gut microbiota, increasing *Ligilactobacillus* abundance and shifting arginine and proline metabolism. In female mice, Tau maintained circulating estrogen levels, which in turn upregulated AICDA expression and enhanced IgA-mediated intestinal immunity. Under the specific dosing regimens and radiation parameters utilized in this study, Tau neither altered baseline tumor progression nor compromised the tumor response to irradiation. We conclude that Tau protects against RIII through sex-specific mechanisms, microbiota reprogramming in males and estrogen-AICDA-IgA signaling in females, without compromising the antitumor efficacy of radiotherapy.

## 1. Introduction

Radiotherapy is a cornerstone in the treatment of abdominal and pelvic malignancies, but its use is often dose-limited by collateral damage to adjacent healthy intestines, leading to RIII 1. The pathophysiological features of RIII include intestinal stem cell apoptosis, disruption of crypt-villus architecture, loss of tight junction proteins, and breakdown of the mucosal barrier 2, 3. In addition, ionizing radiation (IR) induces severe intestinal dysbiosis and local immune disturbances, which further promote inflammatory cascades 4, 5. Clinically, RIII presents as severe diarrhea, intestinal perforation, and systemic infections, impairing patients' quality of life 6, 7. Despite the clear clinical need for interventions against RIII, specific preventive measures remain very limited. Traditional symptomatic treatments are often ineffective, and existing radiation protectors carry theoretical flaws, as they may also shield tumor tissues 8-10. Therefore, how to effectively protect the normal intestine without compromising the anti-tumor effect remains an urgent problem to be solved.

Taurine (Tau) is an endogenous sulfur-containing amino acid with antioxidant, anti-inflammatory, and osmoregulatory abilities 11-13. However, how it shields the intestine from IR-induced damage, and the mechanism, are still unclear. Studies suggest sex affects radiation sensitivity and gut microbiota composition 14, 15. Notably, the radioprotective efficacy of certain agents may be sex-dependent, a factor that has received little attention in current research.

This study assessed the radioprotective effects of Tau in mice models of WAI, with a focus on sex-dependent mechanisms. We found two different pathways at work. In males, the protection involved a microbiota-metabolome axis; in females, an estrogen-mucosal immune axis. Specifically, Tau helped maintain circulating estrogen levels in female mice and, through the estrogen-AICDA axis, boosted the IgA-dependent intestinal immune barrier. We also tested the oncological safety of Tau in several *in vivo* tumor models. In all cases, Tau neither accelerated baseline tumor growth nor shielded malignant cells from radiation therapy. Thus, as an inexpensive, well-tolerated, endogenous amino acid, Tau administered in a sex-specific manner provides a mechanistically distinct radioprotective adjunct with translational potential.

## 2. Materials and Methods

### 2.1 Animals and ethical approval

Male and female wild-type C57BL/6J and BALB/c (nu/nu) mice (6-8 weeks old) were purchased from Beijing HFK Bioscience Co., Ltd. (Beijing, China). The animals were maintained under specific pathogen-free (SPF) conditions at the Institute of Radiation Medicine, Chinese Academy of Medical Sciences (IRM-CAMS), with male and female mice housed in the same room, a 12 h/12 h light/dark cycle, and free access to standard diet and water. All animals received identical batches of basal diet throughout the trial to eliminate dietary-induced fluctuations in baseline arginine and proline metabolism. Treatment and control cohorts were housed in independent cages to avoid microbial cross-contamination. After a seven-day adaptive acclimatization, all experimental mice were assigned to different groups using a standard random number table to eliminate selection bias and ensure balanced sample distribution across all treatment and control cohorts. To minimize cage-related artifacts, mice from each experimental group were evenly distributed across multiple breeding racks with randomized cage placement. All animal experiments conducted in this study were carried out in accordance with a protocol approved by the Institutional Animal Care and Use Committee of IRM, CAMS.

### 2.2 Whole-abdominal irradiation and treatment

Irradiation was performed using a ¹³⁷Cs γ-ray irradiator (Gammacell-40; Atomic Energy of Canada Ltd., Chalk River, ON, Canada) with a calibrated dose rate of 0.8 Gy/min. Prior to irradiation, animals were anesthetized with isoflurane and then subjected to WAI at specified doses. During exposure, the thorax, head, and limbs were shielded with custom lead blocks. Control animals underwent a sham-irradiation procedure. Mechanistic evaluations were performed uniformly at 12 Gy WAI in both sexes, whereas the sex-specific doses of 13 Gy (males) and 15 Gy (females) were reserved solely for survival assays to achieve comparable baseline lethality. The rationale for applying sex-specific radiation doses is based on the inherent differences in radiation tolerance between sexes. These specific doses were previously determined and validated in our established studies to account for these physiological disparities and to construct an optimal injury model 16, 17. Tau (Aladdin, China) was dissolved in distilled water and administered via oral gavage at a dosage of 1000 mg/kg 12, and this dose has been rigorously validated in previous studies and has been shown to exert effective antioxidant and tissue-protective effects in mouse models without causing physiological toxicity. The first dose was given 1 h before irradiation, followed by daily doses for three consecutive days post-exposure.

### 2.3 Ovariectomy (OVX) and 17β-estradiol (17β-E2) supplementation

Female C57BL/6J mice were randomly assigned to OVX or OVX with 17β-E2 (HY-B0141, MedChemExpress) supplementation (OVX+17β-E2). After one week of acclimatization, both groups underwent surgery. Briefly, under isoflurane anesthesia, a ventral midline incision was made to expose the abdominal wall. The ovaries were located, gently retracted, and excised near the uterine horns 18. For continuous hormone replacement in the OVX+17β-E2 group, slow-release capsules were fabricated using 2-cm segments of silastic tubing. One end of each tube was sealed with a 3-mm wooden plug, and the capsules were filled with a 30 μg/mL 17β-E2 in sesame oil. The open end was then sealed with a second wooden plug. The filled capsules were incubated overnight in the same estradiol solution and subsequently implanted subcutaneously into the mice 19.

### 2.4 Histopathological and immunohistochemical (IHC) analyses

To evaluate mucosal architecture and Tau -mediated cyto protection, on day 5 post-WAI, the small intestine, colon, and Peyer's patches (PPs) were harvested and immediately fixed in 4% paraformaldehyde. Tissue samples were fixed in 4% paraformaldehyde, paraffin-embedded, and sectioned at 3-5 μm thickness. Sections were stained with hematoxylin and eosin (H&E) and periodic acid-Schiff (PAS) according to standard protocols. For IHC, sections were deparaffinized, rehydrated, and subjected to antigen retrieval, followed by overnight incubation at 4°C with primary antibodies against AID (392500; Invitrogen), CD45R (12-0452-82; eBioscience), Villin (ab130751; Abcam), Ki67 (ab15580; Abcam), MUC2 (GB11344; Servicebio), ZO-1 (GB111402; Servicebio), Occludin (GB111401; Servicebio), and F4/80 (GB113373; Servicebio). After washing, sections were incubated with corresponding biotinylated secondary antibodies. All stained sections were digitally scanned using a Pannoramic P250 scanner (3DHISTECH, Hungary) and analyzed using CaseViewer software. All histological slides and imaging files were labeled with anonymous unique identifiers to mask group information. Histopathological scoring and image quantitative analysis were independently completed by two investigators who were fully blinded to the experimental grouping.

### 2.5 Enzyme-linked immunosorbent assay (ELISA)

Peripheral blood was gathered from the orbital sinuses, then centrifuged at 4,000 rpm for 10 min at 4 °C to obtain serum. Following the product instructions, concentrations of IL-1β, IL-6, and TNF-α were measured using specific ELISA kits (Tongwei, China). Circulating 17β-E2 levels were determined using a commercial ELISA kit (501890; Cayman Chemical). All procedures followed the manufacturers' instructions.

### 2.6 Bacterial diversity analysis

To evaluate the impact of WAI and Tau treatments on gut microbiota composition, we collected fresh fecal samples for 16S rRNA gene sequencing analysis. DNA was isolated via the CTAB/SDS method, followed by amplification of the V4 hypervariable region after quality control. Sequencing libraries were prepared and run on an Illumina NovaSeq platform (Novogene Bioinformatics Technology Co., Ltd., Beijing, China). Using QIIME2, we assigned taxonomy and aligned sequences. Alpha and beta diversity indices were calculated based on normalized read counts, with beta diversity assessed using the unweighted UniFrac distance metric at the OTU/ASV level and visualized via principal coordinate analysis (PCoA). Differential features were identified using LEfSe, with a logarithmic LDA score threshold of > 2.0 and a significance level of p < 0.05 17.

### 2.7 Untargeted metabolomic relative-quantitative analyses

Untargeted metabolomic profiling was conducted on a UHPLC-HRMS platform (Novogene Co., Ltd., Beijing, China) under positive and negative ionization modes. Pooled quality control (QC) samples were run periodically to monitor instrument stability. Raw data were converted to mzXML format using ProteoWizard and then processed with XCMS for peak detection, alignment, and retention time correction. Metabolic features with >50% missing values in any group were excluded, and the remaining peak areas were normalized to the total integrated area per sample. Metabolite annotation was performed by matching MS/MS fragmentation patterns and isotopic distributions to an in-house database and public repositories. Features with QC coefficient of variation (CV) < 30% were retained for multivariate analysis, including principal component analysis (PCA) and partial least squares discriminant analysis (PLS-DA). Significantly altered metabolites were identified with a composite threshold of variable importance in projection (VIP) > 1 and fold change > 2. Finally, Pathway enrichment analysis was performed using KEGG to characterize the biological functions and topological networks of the identified metabolites 16.

### 2.8 RNA-seq

Total RNA extracted from small intestinal tissues was assessed for integrity. Library preparation and paired-end sequencing were performed on an Illumina platform (Novogene Bioinformatics Technology Co., Ltd., China). Raw reads were subjected to quality filtering and normalization, followed by principal component analysis (PCA) for dimensionality reduction. Inter-sample reproducibility was evaluated using Pearson correlation analysis. Gene Ontology (GO) and Kyoto Encyclopedia of Genes and Genomes (KEGG) enrichment analyses were performed to identify molecular pathways associated with irradiation and taurine treatment, with significance set at *p* < 0.05. For GSEA, pathways with a nominal *p* < 0.05 and a normalized enrichment score (NES) with an absolute value > 1.0 were considered significantly enriched.

### 2.9 Flow cytometry analysis

Lymphocytes were isolated from PPs to generate single-cell suspensions as previously described 20. Prior to surface labeling, Fc receptors were blocked utilizing a CD16/32 antibody (14-9161-73; eBioscience) to prevent non-specific binding. Cells were then surface stained with anti-CD19 (6D5; 115541; BioLegend). Following surface phenotyping, cells were fixed and permeabilized using BD Cytofix/Cytoperm buffer at 4 °C for 20 min. After two washes with BD Perm/Wash buffer, cells were resuspended in Hank's Balanced Salt Solution supplemented with 1% BSA and incubated overnight at 4 °C. Intracellular staining was subsequently performed with anti-IgD (405714; Biolegend), anti-CD138 (142530; Biolegend), anti-IgA (12-4204-82; Invitrogen), and AID-biotin (mAID-2; 13-5959-82; eBioscience), followed by incubation with FITC-conjugated streptavidin (405201; BioLegend). Data were acquired on a FACSC elesta flow cytometer (BD Biosciences) and analyzed utilizing FlowJo software (Tree Star). Flow cytometry data acquisition was performed by investigators blinded to the group allocation.

### 2.10 Intestinal organoid culture

Murine intestinal crypts were isolated, cultured, and passaged to generate organoids following established protocols 17. To test how well Tau protects intestinal stem cells (ISCs) from radiation outside the body, we resuspended the isolated cells in Matrigel (Corning) and seeded them into 48-well plates. Complete IntestiCult™ Organoid Growth Medium (STEMCELL Technologies) was applied to each well. To validate mechanisms under the IR + Tau + 17β-E2 condition, 17β-E2 was added to the culture medium. Cells were exposed to Tau for 30 min prior to irradiation at 5 Gy. Bright-field microscopy was used to monitor organoid morphogenesis.

### 2.11 Cell culture and clonogenic assays

Normal human intestinal epithelial cells (HIEC-6), murine small intestinal epithelial cells (MODE-K), and murine colon carcinoma cells (MC38) were maintained in RPMI 1640 medium (Invitrogen) supplemented with 10% fetal bovine serum (FBS). Human colorectal carcinoma HCT-116 cells and cervical epidermoid carcinoma Me-180 cells were cultured in McCoy's 5A medium containing 10% FBS. All cell lines were grown at 37 °C in a humidified 5% CO₂ atmosphere. For clonogenic survival assays, HIEC-6 and MODE-K cells were seeded into 6-well plates at a density of 1,000 cells/well. Cells were pretreated with either vehicle or 10 nM Tau for 30 min, followed by exposure to 5 Gy irradiation. After 12 days of incubation, colonies were fixed, stained with 0.1% crystal violet, and those containing ≥ 50 cells were counted.

### 2.12 Primary AOM/DSS-induced colorectal cancer model

We established a colitis-associated colorectal cancer model using azoxymethane/dextran sulfate sodium (AOM/DSS) Mice received a single intraperitoneal injection of AOM (10 mg/kg; Sigma-Aldrich, A5486). Tumor promotion was achieved through three consecutive cycles of 2% (w/v) DSS in drinking water for 5 days, each followed by 14 days of regular distilled water. After tumor induction, mice in the WAI and WAI+Tau groups received WAI at 12 Gy. Tau (1000 mg/kg) was administered by oral gavage starting 1 hour before WAI and continued for three consecutive days post-WAI. Small intestinal and colonic tissues were subsequently collected for macroscopic and molecular analyses.

### 2.13 Subcutaneous tumor xenograft models

To assess whether Tau modulates tumor radiosensitivity, subcutaneous xenograft models were established. HCT-116 cells (4 × 10⁶) were injected into the right dorsal flank of male BALB/c-nu/nu mice, Me-180 cells (4 × 10⁶) into female nude mice, and MC38 cells (1 × 10⁶) into male and female C57BL/6J mice. When mean tumor volumes reached approximately 100 mm³ (calculated as length × width² × 0.5), mice from each sex and cell line were randomly assigned to four groups (n = 5): Control, Tau, IR, and IR+Tau. Tau was administered daily by oral gavage as described previously. Mice in the IR and IR+Tau groups received fractionated focal IR (4 Gy/day for three consecutive days; cumulative dose 12 Gy) directed at the tumor bed, with the rest of the body shielded by lead blocks. Control and Tau-only mice underwent sham irradiation. Tumor dimensions were measured every 4 days using calipers.

### 2.14 Statistical analysis

All statistical analyses were executed utilizing GraphPad Prism 9 software. The significance of differences among multiple groups was determined using a one-way analysis of variance (ANOVA). Survival outcomes were plotted using the Kaplan-Meier method and statistically compared, the body weight comparison of surviving mice was conducted using the dual tailed Mann Whitney U test. Data are uniformly presented as the mean ± standard deviation (SD). A *p*-value of < 0.05 was considered to indicate statistical significance.

## 3. Results

### 3.1 Taurine protects against WAI-induced lethality and preserves intestinal stem cells function

To investigate the radioprotective effects of Tau, male and female mice were subjected to 13 Gy and 15 Gy WAI, respectively (Fig. 1A, D). Over a 30-day post-WAI period, the survival rates of control male and female mice were 50%, whereas Tau-treated males achieved 91.7%. Similarly, control female mice had a survival rate of 66.7%, and Tau treatment increased this to 100% (Fig. 1B, E). A clear sex difference emerged in how body weight changed over time in response to Tau supplementation. In females, the IR+Tau group weighed significantly more at Day 9 (raw *p* = 0.0083, FDR-adjusted Q = 0.0913) and Day 24 (raw *p* = 0.0096, Q = 0.0528), with a near-significant difference still present at Day 30 (*p* = 0.0627). In males, no early effect appeared (Day 9, *p* = 0.2143); rather, differences arose from Day 15 onward, with the strongest signals at Days 15, 24 and 27 (raw *p*-values: 0.0203, 0.0497, and 0.0089; corresponding Q-values 0.1117, 0.1822, and 0.0979). By Day 30, the male difference had nearly vanished (*p* = 0.2079), whereas the female difference persisted marginally (*p* = 0.0627). Following FDR adjustment, however, none of these comparisons reached statistical significance. Nevertheless, the contrasting temporal profiles, early and sustained in females versus delayed, pulse-like (peaking at Day 27 and then rapidly waning) in males, point to a sex-specific, time-constrained effect of taurine on post irradiation weight dynamics (Fig. 1C, F). We next evaluated the in vitro radioprotective efficacy of Tau using HIEC-6 and MODE-K intestinal epithelial cell lines. Clonogenic assays showed that Tau treatment markedly enhanced the survival and proliferative capacity of irradiated cells (Fig. 1G-I). We then examined the protective effect of Tau on ISCs. The small intestinal crypts isolated from mice gave rise to budding structures by day 4 of culture and gradually matured into complex, multi-lobed organoids (Fig. 1J). IR greatly lowered the efficiency of organoid formation. In contrast, Tau pretreatment boosted the survival of intestinal stem cells after IR (Fig. 1K, L). H&E and Ki67 staining confirmed that Tau reduced irradiation-induced structural damage and maintained proliferative activity in the organoids (Fig. 1M). In summary, Tau effectively protects against radiation-induced intestinal injury, significantly improves post-irradiation survival in mice, and preserves the regenerative capacity of intestinal epithelial cells and crypts.

### 3.2 Taurine mitigates WAI-induced intestinal injury and systemic inflammation in mice

To evaluate the protective efficacy of Tau against radiation-induced intestinal damage, male and female mice were exposed to 12 Gy WAI (Fig. 2A). WAI induced marked intestinal hyperemia and edema, which were significantly attenuated by Tau treatment (Fig. 2B, D). Concurrently, Tau mitigated the severe body weight loss observed in irradiated mice (Fig. 2C, E, and Fig. S1A, B). WAI also caused dramatic colonic shortening, whereas Tau administration preserved colonic length close to normal levels (Fig. 2F-I). Fecal output monitored over a 30 min period prior on Day 3 pose-WAI revealed that Tau effectively prevented the IR-induced reduction in defecation (Fig. 2J). In addition, Tau partially reversed the irradiation-driven atrophy of the spleen and thymus (Fig. S2A-H). Histopathological analysis showed that WAI caused disruption of the intestinal villi, loss of crypt structure, and a reduction in goblet cells in both male and female mice. In contrast, Tau-treated mice retained a more intact crypt-villus architecture and normal goblet cell numbers (Fig. 2K, L). IHC analysis suggested that Tau restored the expression of MUC2 and the tight junction protein ZO-1, both vital for the intestinal barrier (Fig. 2M, N) 21, 22. Additionally, Tau markedly suppressed IR-induced inflammation, lowering the levels of IL-6, IL-1β, and TNF-α (Fig. 2O-T). Collectively, Tau effectively alleviates WAI-induced intestinal damage and the associated inflammatory response in mice.

### 3.3 Sex-dependent regulation of the intestinal microbiota by Taurine after WAI

To investigate sex-dependent mechanisms of Tau-mediated radioprotection, we performed 16S rRNA gene sequencing on fecal samples from male and female mice after WAI. For clarity, all differential microbial changes in male mice are presented consistently at the genus level. In male mice, alpha-diversity analysis showed that Tau altered gut microbial community structure. Chao1 estimates richness, Shannon reflects richness and evenness, Simpson captures dominance/evenness, and Dominance indicates whether a few taxa disproportionately prevail. Tau increased community diversity without markedly affecting overall richness in male mice (Fig. 3A-D). In contrast, no such changes were observed in female mice (Fig. 3E-H). Normalized stochasticity ratio (NST) analysis showed that Tau had a stronger modulatory effect on the gut microbiome in irradiated males than in females (Fig. 3I-J). Principal coordinate analysis (PCoA) revealed that Tau treatment induced a clear separation in microbial composition from irradiated controls in both sexes (Fig. 3K-L). To confirm the statistical significance of this visual separation, PERMANOVA (Adonis) was performed. The analysis demonstrated highly significant differences in beta-diversity community structures between the WAI and WAI+Tau groups in both male (R^2^ = 0.198, *p* = 0.001) and female (R^2^ = 0.161, *p* = 0.001) cohorts. LEfSe cladograms revealed irradiation-induced dysbiosis at multiple taxonomic levels. After Tau administration, the disrupted microbial structure recovered more clearly in male mice (Fig. 3M-N). Genus-level clustering heatmaps further showed that certain bacterial taxa altered by irradiation returned toward baseline levels after Tau treatment (Fig. S3A-B). Inter-group species analysis (t-test) further showed that taurine increased the abundance of *Ligilactobacillus* in the intestines of both male and female mice, while reducing that of *Bacteroides* (Fig. 3O-P). However, there was no significant difference in the baseline abundance of *Ligilactobacillus* and *Bacteroides* at the genus level between untreated male and female mice (Fig. S3C). Collectively, Tau modulates the post-irradiation gut microbiota in a sex-dimorphic manner, with greater efficacy in restoring microbial structure and diversity in males. This suggests that the gut microbiota may mediate the sex-based differences observed in Tau-conferred radioprotection.

### 3.4 Taurine confers radioprotection through sex-dependent remodeling of *Ligilactobacillus* and arginine-proline metabolism

Alterations in the gut microbiota are intrinsically linked to dynamic shifts in their metabolic output 23. To delineate the underlying mechanisms of Tau-mediated radioprotection, we comprehensively evaluated the intestinal metabolomic profiles of irradiated mice. Volcano plot analysis revealed that, relative to controls, male mice in the WAI group exhibited 482 upregulated and 660 downregulated metabolites. Following Tau administration, 1073 metabolites were upregulated while 296 were downregulated compared to the WAI group (Fig. 4A-B). In female cohorts, WAI induced the upregulation of 426 and downregulation of 1183 metabolites, whereas Tau intervention resulted in 867 upregulated and 124 downregulated metabolites (Fig. 4C-D). Three-dimensional principal component analysis (3D-PCA) suggested a distinct segregation between the WAI and control groups (Fig. S4A-B), and the metabolomic signatures of the Tau-treated mice also diverged sharply from those of the WAI model group (Fig. 4E-F). Moreover, subcluster analysis confirmed that Tau effectively reversed the aberrant fluctuations of various metabolite clusters induced by WAI (Fig. 4G-H). Gene Set Enrichment Analysis (GSEA) indicated that the arginine and proline metabolism pathway was significantly enriched in Tau-treated irradiated male mice. Corresponding clustering heatmaps visually validated the elevated abundance of key metabolites within this pathway (Fig. 4I-J). Biologically, arginine and proline are highly versatile amino acids that act as essential precursors for polyamine biosynthesis and collagen deposition, both of which are strictly required for rapid mucosal repair and enterocyte proliferation following radiation damage 24. Conversely, this metabolic pathway was not significantly enriched in female mice, lacking consistent alterations in the related metabolites (Fig. 4K-L). Although this specific pathway was not the most prominent in conventional KEGG enrichment analyses (Fig. S4C-D), the distinct sex-dimorphic microbial regulation by Tau led us to investigate a functional link between core taxa, such as *Ligilactobacillus*, and arginine and proline metabolism. Consequent integrative analyses between the microbiome and metabolome unveiled a strong positive correlation between the abundance of *Ligilactobacillus* and multiple metabolites within this pathway in male mice. In stark contrast, despite being modulated by Tau, *Ligilactobacillus* exhibited no significant correlation with these metabolites in females (Fig. 4M-N and Fig. S4E-F). Collectively, these findings suggested that Tau regulates the intestinal microecology in a sex-dependent manner, achieving its radioprotective efficacy in male mice primarily through the concerted remodeling of *Ligilactobacillus* and arginine-proline metabolism.

### 3.5 Taurine modulates the intestinal immune network for IgA production in a sex-dependent manner via *AICDA*

Tau treatment induced significant gut microbial and metabolic remodeling in irradiated male mice, whereas no obvious microbial alteration was observed in female counterparts. We therefore further performed transcriptomic profiling to explore the unique taurine-mediated radioprotective mechanism in females. Pearson correlation analysis verified high transcriptomic consistency among biological replicates within each group (Fig. S5A-B). In male mice, WAI induced the upregulation of 356 genes and downregulation of 451 genes relative to controls. Subsequent Tau intervention yielded 372 upregulated and 327 downregulated genes compared to the WAI group (Fig. 5A-B). In the female cohort, WAI resulted in 128 upregulated and 783 downregulated genes, whereas Tau administration reversed this trend, upregulating 320 and downregulating 140 genes (Fig. 5C-D). Gene Ontology (GO) enrichment analysis revealed that differentially expressed genes (DEGs) induced by WAI in male mice were primarily associated with T cell activation and localized to side of membrane (Fig. S5C-D). Following Tau treatment in males, DEGs were predominantly enriched in positive regulation of antigen receptor-mediated signaling pathway and structural components such as the cell-cell junction (Fig. 5E-F). In female mice, IR profoundly impacted adaptive immunity and B cell activation (Fig. S5E-F); conversely, Tau specifically modulated genes governing B cell activation and immune responses (Fig. 5G-H). While WAI universally triggered GO shifts tied to general immunological collapse, Tau drove female-specific DEGs toward the positive regulation of antigen receptor signaling and B cell activation. Further pathway analysis utilizing the KEGG database underscored the sex-dimorphic effects of Tau. WAI disrupted multiple immune pathways, including “Intestinal immune network for IgA production”, “Hematopoietic cell lineage”, and “Primary immunodeficiency” pathway, in both sexes. Tau administration selectively enriched these pathways in female mice but not in males (Fig. 5I-M). To pinpoint the exact target mediating this protection, we focused on the “Intestinal immune network for IgA production” pathway. Differential expression profiling within intestinal immune network for IgA production pathway highlighted that* AICDA* was the most sensitively responsive and significantly upregulated gene following Tau treatment (Fig. 5N). Taken together, these findings suggest that Tau exerts female-specific radioprotective effects, with AICDA acting as a possible mediator in promoting the recovery of the intestinal immune network for IgA production.

### 3.6 Taurine-mediated radioprotection of mucosal immunity is dependent on ovarian estrogen *in vivo*

RNA-seq profiling singled out AICDA as a central regulator underlying the sex-specific radioprotective effect of Tau. Since AICDA transcription is directly upregulated by estrogen signaling 25, 26, we hypothesized that this sex-dependent protection relies on ovarian hormones. To test this, we employed an OVX mouse model subjected to WAI (Fig. 6A). Serum analysis confirmed that WAI moderately decreased estradiol levels, whereas Tau administration (WAI+Tau) partially maintained them. As expected, OVX severely depleted serum estradiol levels, and exogenous 17β-E2 supplementation successfully restored them to physiological levels (Fig. 6B).

Flow cytometry was performed to assess mucosal immune cell populations. WAI reduced the percentages of AICDA⁺CD19⁺ B cells (Fig. 6C, D) and IgA⁺CD138⁺ plasma cells (Fig. 6E, F) in ovary-intact mice. Tau treatment restored these populations in ovary-intact mice, but this effect was absent in OVX mice. Administration of 17β-E2 to OVX mice treated with WAI and Tau restored the protective effect of Tau, increasing both AICDA⁺ B cells and IgA⁺ plasma cells. Immunofluorescence staining of lymphoid tissues confirmed that WAI disrupted tissue structure and reduced AICDA⁺ and B220⁺ cells. Tau preserved these structures, an effect that was abolished by OVX and reinstated by 17β-E2 (Fig. 6G). *In vitro* organoid formation assays showed that WAI impaired organoid formation, and Tau promoted morphological recovery after irradiation. Combined treatment with Tau and 17β-E2 produced a protective effect similar to that of Tau alone, with no additional enhancement (Fig. 6H-I). These results suggested that while Tau alone can help epithelial organoids recover *in vitro*, but in living animals, estrogen is absolutely needed for Tau to protect against immune damage caused by IR.

### 3.7 Taurine protects normal tissues without compromising tumor radiosensitivity or promoting tumor growth

The above results show that Tau mitigates radiation-induced intestinal injury in a sex-dependent manner. To evaluate the safety of Tau in the context of radiotherapy, primary colorectal cancer models were established in both male and female mice using AOM/DSS (Fig. 7A). After WAI and Tau administration, radiation-induced colonic shortening in normal tissue segments was reversed (Fig. 7B-C). Macroscopic examination of opened colons and Ki67 staining of tumor tissues indicated that Tau alone did not promote baseline tumor progression. WAI suppressed tumor growth, and concurrent Tau administration did not reduce tumor radiosensitivity (Fig. 7D-G). To rule out potential confounding effects from the tumor microenvironment, we established subcutaneous MC38 xenografts in both male and female mice (Fig. 7H-I). After local irradiation and Tau administration, we found that Tau had no effect on baseline tumor progression or radiation response (Fig. 7J-L and Fig. S7A-D). To further validate the effects of Tau across different tumor types, we tested human colon cancer HCT116 cells using the same approach. Given that Tau was found to maintain circulating estrogen levels, we also examined its effects on a female-derived cervical cancer cell line Me 180. In all cases, Tau did not affect tumor growth or sensitivity to radiotherapy (Fig. 7M-O and Fig. S7E-H). Collectively, these protective effects occur only in normal tissues, with no impact on baseline tumor progression or the tumor response to irradiation that we studied.

## 4. Discussion

Tau modulates post-irradiation intestinal homeostasis in a sex-dependent manner. While it protects against radiation-induced intestinal injury in both sexes, the primary regulatory pathways exhibit distinct sex-biased tendencies: a more prominently associated microbiota-metabolite axis in males and an estrogen-mucosal immune axis in females. Importantly, it should be noted that these pathways represent predominant sex-biased tendencies rather than absolute sex-exclusive mechanisms. Biological radiation injury repair and small-molecule-mediated intestinal protection are rarely completely segregated by sex. While our data suggest that the microbiota-metabolic axis is more prominent in males and the estrogen-immune axis dominates in females, both regulatory networks likely exist across sexes with differential magnitudes. Therefore, the sex-dependent protective responses observed highlight a difference in the primary dependency of these pathways rather than a complete absence in the opposite sex. This divergence is consistent with established sex differences in responses to genotoxic stress 27, 28. Together, these findings link systemic endocrine signaling to gut microbial ecology and provide a mechanistic basis for the sex-stratified use of Tau as a radioprotectant.

In male mice, the radioprotective efficacy of Tau was strongly associated with the gut microbiota. Tau intervention alleviated IR-induced gut microbiota imbalance, with a particularly notable increase in *Lactobacillus* 29, 30. It has been established that specific commensal bacteria can attenuate radiation toxicity through the secretion of protective metabolites 31. Consistent with this, the results of the experimental genomics indicate that the changes in microbial composition were closely linked to alterations in arginine and proline metabolism. These two amino acids serve as precursors for glutathione and polyamine biosynthesis. Polyamines play a crucial role here, they mop up reactive oxygen species (ROS) and help the gut lining recover after IR damage 24, 32. Collectively, these data indicate that, in male mice, Tau may contribute to the antioxidant defense of the intestinal crypts via targeted reshaping of the gut microbiota.

In female mice, the gut microbiota is dispensable for the radioprotective effect of Tau, which instead depends on the crosstalk between endocrine and mucosal immune systems. As the ovary is highly sensitive to genotoxic stress, IR-mediated ROS production and DNA damage induce oocyte apoptosis and deplete the ovarian follicular reserve 33 Consequently, the loss of the steroidogenic compartment impairs normal ovarian function and thereby reduces the circulating estrogen levels 34. To preserve this central estradiol axis, Tau likely maintains endocrine homeostasis through multiple mechanisms. By scavenging free radicals to preserve tissue architecture and mitochondrial integrity, Tau directly mitigates radiation-induced follicular damage and protects ovarian function 35. Concurrently, Tau may also indirectly affect endocrine homeostasis through its well-characterized systemic anti-inflammatory effects, preventing radiation-induced inflammatory stress from further disrupting the gonadal axis. Through these combined actions, Tau curtails the extent of injury and successfully maintains normal estradiol levels in IR-exposed females. By binding with estrogen receptors (ERs) in the gut-associated lymphoid tissue, estradiol thereby links the systemic hormonal status to gut immune activity 36. This stable hormonal microenvironment then enhances the AICDA expression of germinal center B cells in PPs 26.

In summary, these results suggest that Tau protects the intestine from IR-induced injury via not only a direct effect on the estrogen-independent intestinal epithelium, but also a systemic estrogen-dependent pathway to shape mucosal immunity. When we cultured organoids with estrogen not being added, the results showed that Tau enhanced the capacity of epithelial cell proliferation and survival after IR. However, we also discovered that the ovariectomy caused a decline of the AICDA⁺ B cells and IgA⁺ plasma cells. This finding is consistent with the female sex hormone preserving mucosal barrier integrity, facilitating B cell maturation, and promoting gut IgA production 37.

These findings should also be interpreted in the context of emerging evidence that biological sex is a critical determinant of radiation response and radioprotective efficacy. Recent studies and reviews have shown that males and females differ in their responses to ionizing radiation at multiple levels, including tissue injury severity, inflammatory and metabolic adaptation, and the efficacy of radiation countermeasures 38. For example, sex-dependent differences have been documented in systemic radiation responses and metabolomic remodeling after irradiation 39. In parallel, recent radiobiology studies have emphasized that the efficacy of radioprotective agents may also vary by sex, further highlighting the need to incorporate sex as a biological variable in radioprotection research 16, 40.

Earlier radioprotection studies were restricted to single agents, mostly in male animals, and rarely considered hormonal changes in females. In contrast, our integrated in vivo model indicates a clearly different result 28. Earlier approaches simply assumed a reduction of oxidative stress within the epithelium should facilitate tissue repair. Here, we show that epithelial survival and mucosal immune recovery are functionally dissociable under Tau treatment. Tau promotes direct protection of the epithelial crypt and villus (evidenced in our *in vitro* studies where 17β-E2 conferred no additional benefit), but does not restore mucosal immune network without estrogen support from other areas of the body. Therefore, the immune modulatory effect of Tau after IR does not appear to be a direct consequence of preserved epithelial barrier; instead, it requires an estrogen dependent mechanism in lymphoid cells. This means that for clinical patients receiving a metabolic radioprotector such as Tau, clinical radiotherapy management should consider ovarian hormone status.

A principal requirement for clinical radioprotector is to spare normal tissues while maintaining efficacy of radiotherapy against tumors 41. In an AOM/DSS-induced primary colorectal cancer model and multiple xenograft models, Tau neither enhanced baseline tumor growth nor reduced tumor radiosensitivity. This ability to protect normal tissues without altering baseline tumor progression or compromising tumor response to irradiation, combined with its endogenous origin, supports exploring Tau as a potential adjunctive agent in clinical oncology.

We should note that radioprotection research up to now has been sex blind, omitting consideration of the obvious biological differences between the sexes. Future work should aim to identify the molecular target of Tau in the ovary setting, and specific bacterial metabolites affecting male intestinal crypts 42. The sex-dependent effects of Tau described here suggest that the response to this radioprotector may involve sex specific mechanisms.

Several limitations and future directions should be acknowledged. First, to bridge the translational gap, future studies must transition from the current single-dose WAI to clinically relevant fractionated irradiation models. Second, the causal contribution of the *Ligilactobacillus* and arginine-proline metabolism axis requires functional validation using targeted approaches, such as fecal microbiota transplantation, mono-colonization, or metabolite interventions. Third, definitively proving the absolute requirement of the downstream estrogen-AICDA-IgA axis presents significant technical hurdles. Achieving localized knockdown of AICDA or IgA in intestinal Peyer's patches without inducing systemic immunodeficiency, which would severely confound radiation injury assessments is exceptionally challenging. Therefore, advanced tools like B-cell-specific conditional knockout models are essential for future validation. Finally, direct causality of female-specific protection should be confirmed via exogenous 17β-E2 supplementation in male mice, while rigorously controlling for hormone-induced microbiome shifts. Identifying the specific mediating estrogen receptor is also necessary for deeper mechanistic insight.

Collectively, Tau protects against RIII via sex-biased mechanisms: In males, protection depends on a microbiota-metabolite pathway; in females, on an estrogen-mucosal immune axis. These data deepen understanding of the biology of RIII and propose a clinically-amenable pathway for our radioprotection. We hope to clinically test these data and move towards personalized radioprotection.

## 5. Conclusion

Overall, we show that the estrogen-AICDA-IgA axis, working alongside specific gut microbiome changes, is the primary driver of female resistance to radiation enteropathy. This finding demonstrates that biological sex is a crucial factor that cannot be ignored when evaluating radiation toxicity and designing protective measures. The interaction between host hormones, immunity, and gut microbes we identified provides a clear direction for future translational research. Moving forward, clinical radioprotective strategies should consider sex differences. Targeting this protective axis through microbiome modulation or manipulation of the AICDA-IgA pathway offers a realistic path toward preventing severe mucosal injury in cancer patients undergoing radiotherapy.

## Supplementary Material

Supplementary figures.

## Figures and Tables

**Figure 1:**
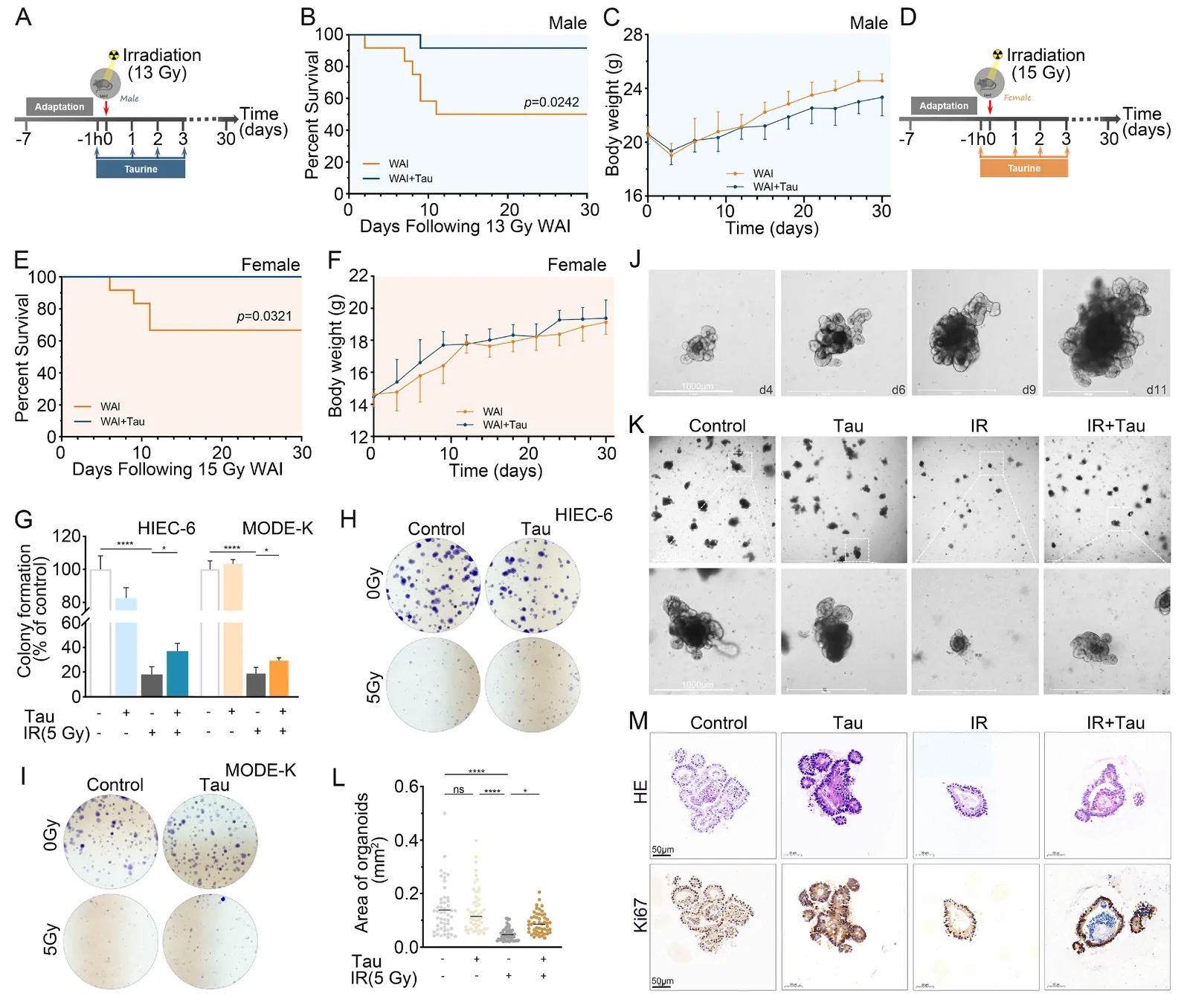
** Tau protects against radiation-induced lethality and preserves ISCs function.** (A, D) Experimental schematics for male (A) and female (D) mice receiving 13 Gy and 15 Gy WAI, respectively. Tau was administered by oral gavage 1 h before irradiation and for three consecutive days following exposure. (B, E) Kaplan-Meier survival curves for male (B) and female (E) mice after WAI (n = 12 per group). (C, F) Body weight changes of male (C) and female (F) mice recorded every 3 days after irradiation (n = 12 per group). (G) Quantitative analysis of colony formation in HIEC-6 and MODE-K cells. (H, I) Representative images of colony formation in HIEC-6 (H) and MODE-K (I) cells. (J) Representative bright-field images of ISC-derived organoids on the indicated culture days. (K) Representative images showing the morphology and size of organoids after one week of culture. (L) Quantification of organoid area. Scatter dots represent individual measurements; error bars indicate 95% confidence intervals (n = 50). (M) Representative H&E and Ki67 staining images of ISC-derived organoids. Data are presented as the means ± SD, ^*^p < 0.05, ^****^p < 0.0001, compared with the indicated groups.

**Figure 2:**
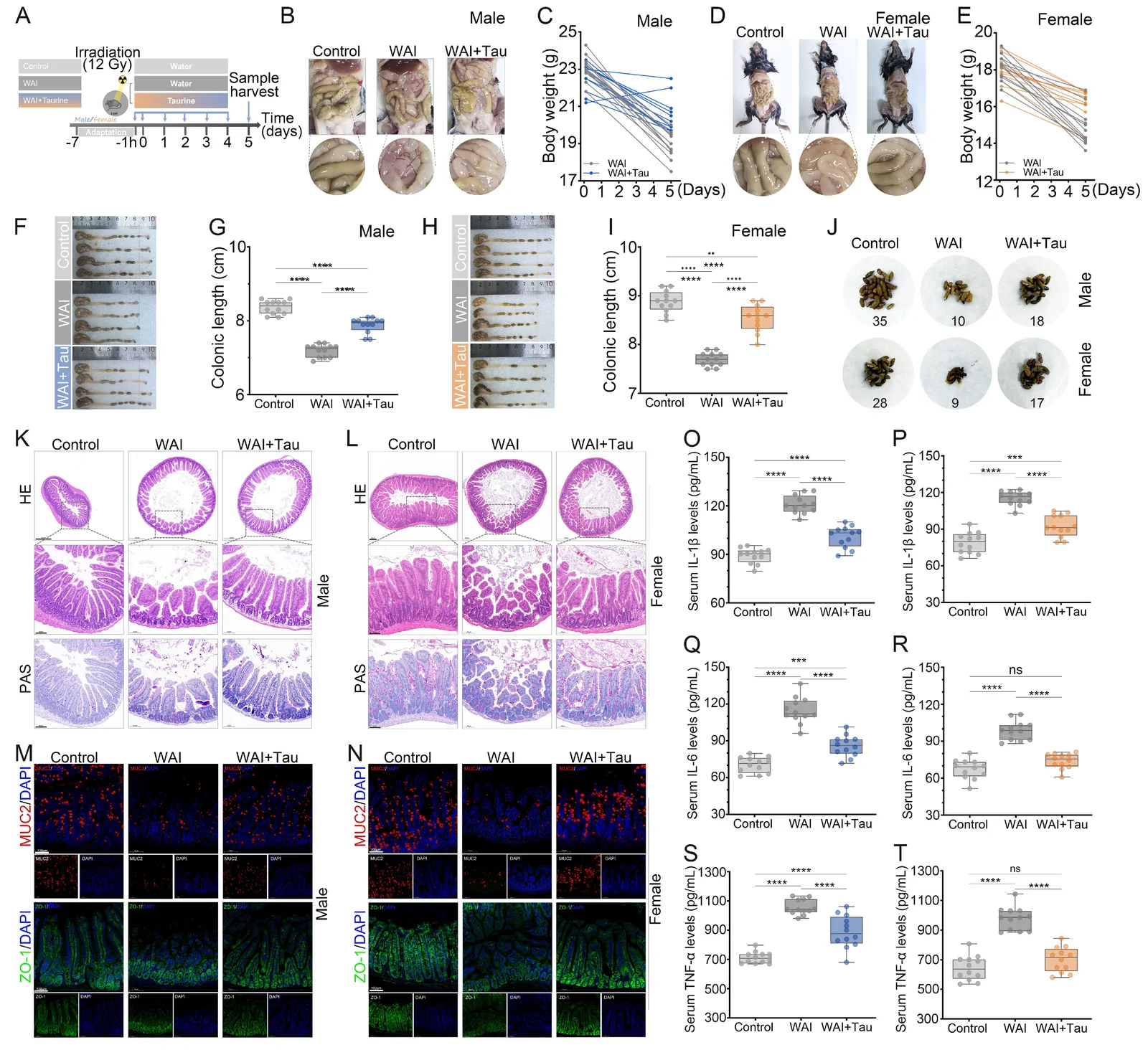
** Tau mitigates WAI-induced intestinal injury and inflammation in mice.** (A) Experimental schematic. Male and female mice were exposed to 12 Gy WAI. Tau was administered by oral gavage1 h before and for three consecutive days after WAI. (B, D) Representative macroscopic images of the gastrointestinal tract in male (B) and female (D) mice. (C, E) Individual body weight trajectories of male (C) and female (E) mice post-IR (n = 12 per group). (F, H) Representative macroscopic images of colons from male (F) and female (H) mice. (G, I) Corresponding quantification of colonic length. (J) Representative images of feces collected over a 30-min period on Day 3 pose-WAI. (K, L) Representative H&E and PAS staining images of small intestinal tissues from male (K) and female (L) mice. Scale bar: 50 µm. (M, N) Representative IHC images of MUC2 and ZO-1 in the small intestines of male (M) and female (N) mice. Scale bar: 50 µm. (O, Q, S) Serum concentrations of IL-1β (O), IL-6 (Q), and TNF-α (S) in male mice (n = 12 per group). (P, R, T) Serum concentrations of IL-1β (P), IL-6 (R), and TNF-α (T) in female mice (n = 12 per group). Data are presented as the means ± SD, ^**^p < 0.01,^ ***^p < 0.001, ^****^p < 0.0001, compared with the indicated groups.

**Figure 3:**
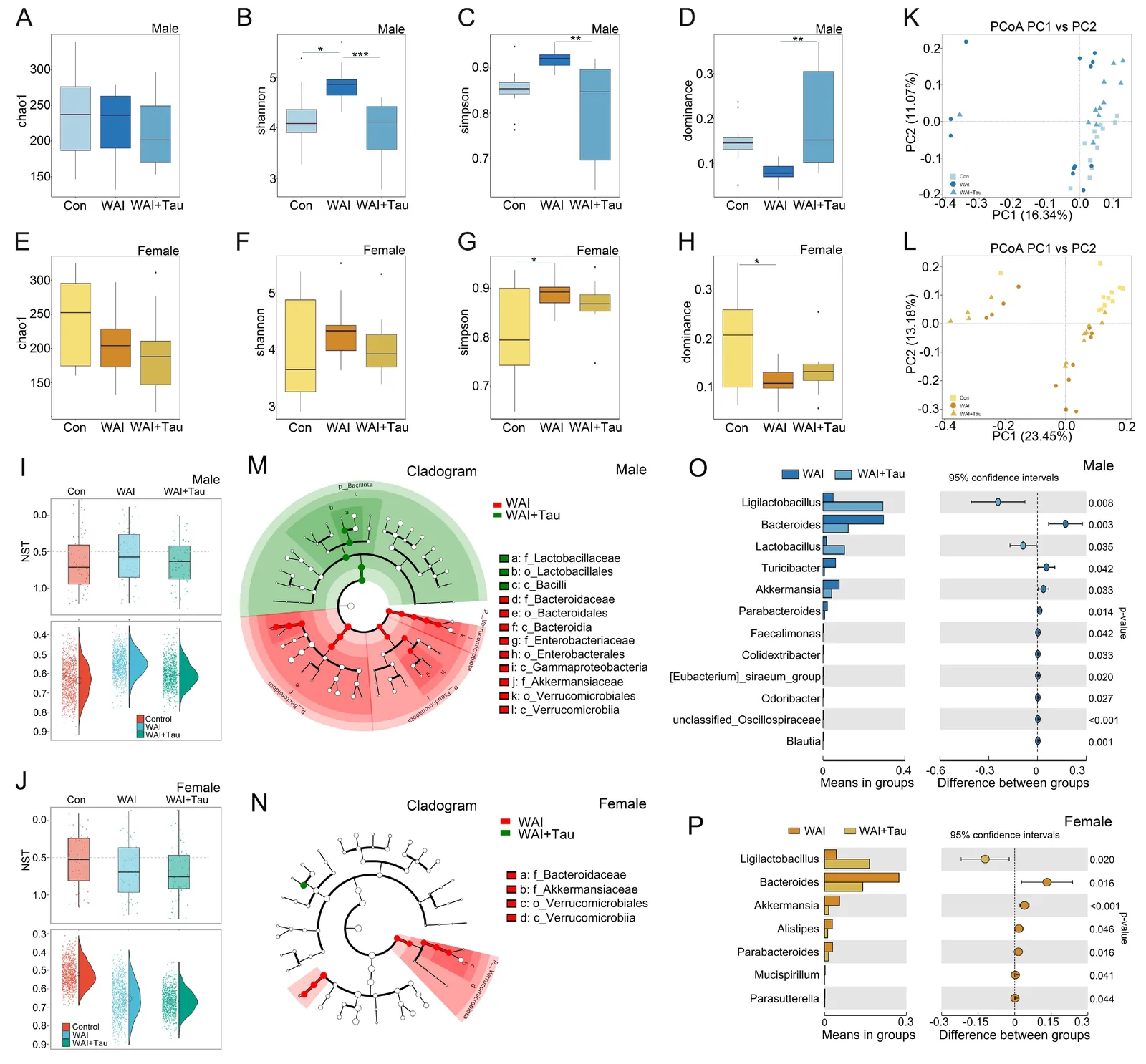
** Sex-dependent changes in post-irradiation gut microbiota following Tau treatment.** (A-D) Alpha diversity metrics (Chao1, Shannon, Simpson, and Dominance indices) for male mice. (E-H) Corresponding alpha diversity metrics for female mice. (I, J) Normalized stochasticity ratio (NST) analysis for male (I) and female (J) groups. (K, L) Two-dimensional principal coordinate analysis (PCoA) plots of gut microbiota composition in male (K) and female (L) mice. (M, N) LEfSe cladograms showing taxonomic differences between experimental groups in male (M) and female (N) mice. (O, P) Inter-group differential taxon analysis (t-test) at the genus level for males (O) and females (P).

**Figure 4:**
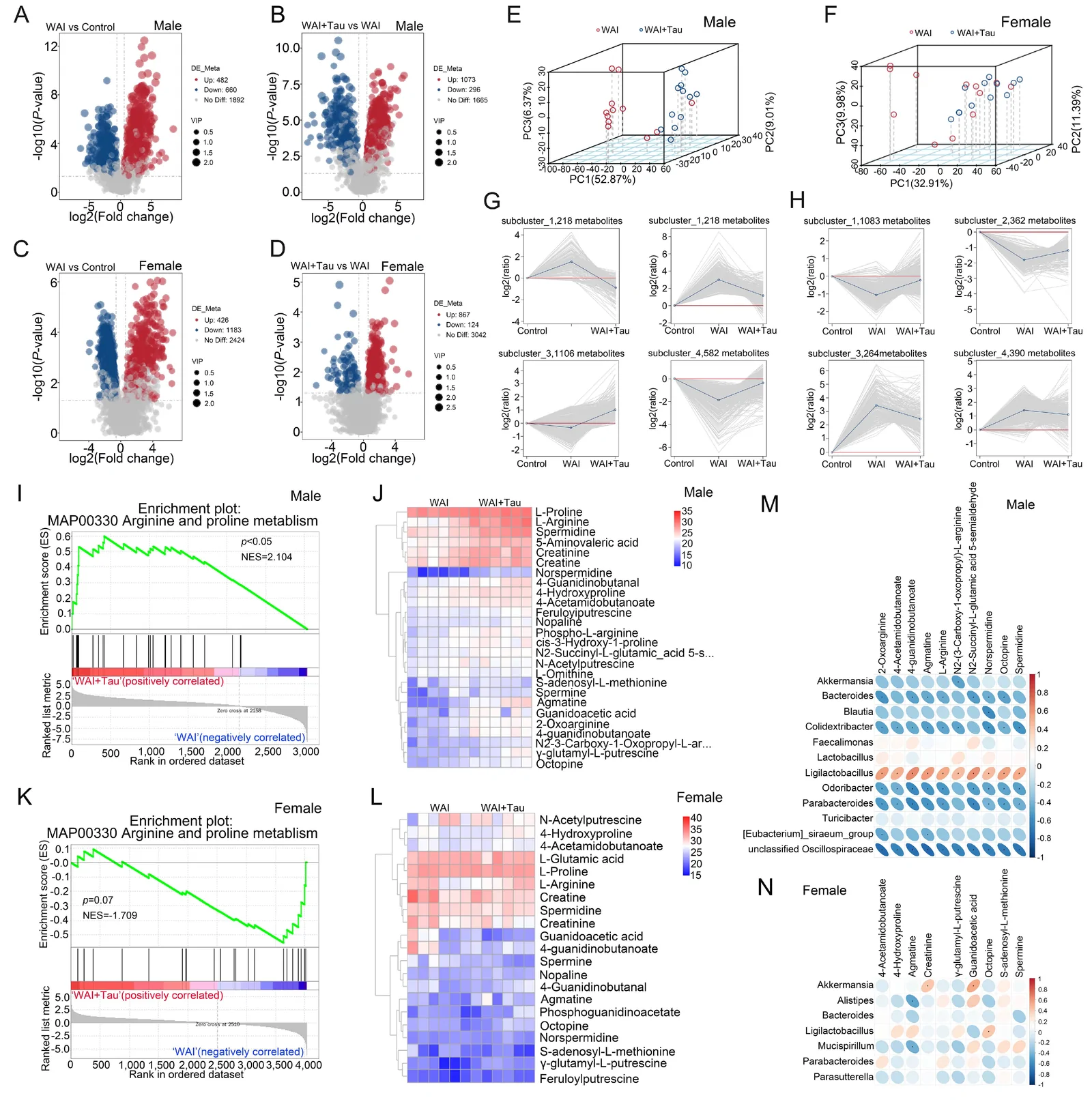
** Sex-dependent modulation of *Ligilactobacillus* and arginine and proline metabolism in Tau-treated irradiated mice.** (A, C) Volcano plots illustrating differentially abundant metabolites between WAI and control groups in male (A) and female (C) mice (*p* < 0.05 and fold change > 2). (B, D) Volcano plots comparing WAI+Tau vs. WAI groups in male (B) and female (D) mice. (E, F) Three-dimensional principal component analysis (3D-PCA) of metabolic profiles comparing the WAI+Tau and WAI groups in males (E) and female (F) mice. (G, H) Subcluster analysis showing abundance trends of distinct metabolite clusters in male (G) and female (H) mice. (I, K) Gene Set Enrichment Analysis (GSEA) of the arginine and proline metabolism pathway in male (I) and female (K) mice. (J, L) Clustering heatmaps of metabolites associated with arginine and proline metabolism in males (J) and females (L) mice. (M, N) Correlation heatmaps showing associations between Tau-modulated gut microbiota and metabolites within the arginine and proline metabolism pathway in irradiated male (M) and female (N) mice.

**Figure 5:**
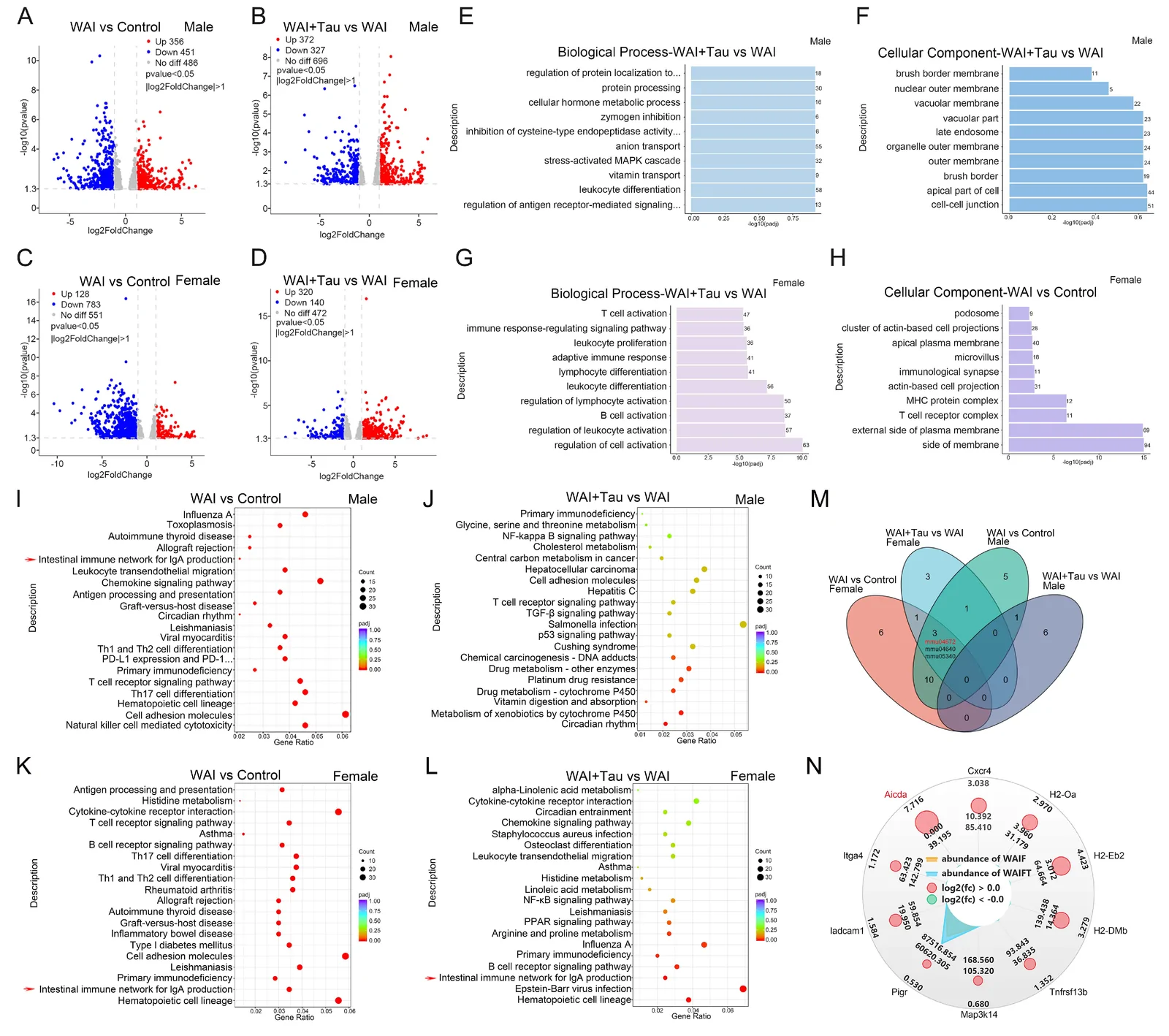
**Tau regulates the intestinal immune network for IgA production in a sex-dependent manner via *AICDA*.** (A, C) Volcano plots of differentially expressed genes (DEGs) between the WAI and control groups in male (A) and female (C) mice. (B, D) Volcano plots of DEGs between the WAI+Tau and WAI groups in male (B) and female (D) mice. (E, G) GO enrichment analysis (Biological Process) for DEGs (WAI+Tau vs. WAI) in male (E) and female (G) mice. (F, H) GO enrichment analysis (Cellular Component) for the same comparisons in male (F) and female (H) mice. (I, K) KEGG pathway enrichment bubble charts comparing the WAI and control groups for male (I) and female (K) mice. (J, L) KEGG enrichment charts for WAI+Tau vs. WAI groups in male (J) and female (L) mice. (M) Venn diagram showing the intersection of the top 10 enriched KEGG pathways across the four comparative groups. (N) Radar-circular plot displaying expression abundance and log2 fold changes of core DEGs within the “Intestinal immune network for IgA production” pathway in female mice; AICDA is highlighted.

**Figure 6:**
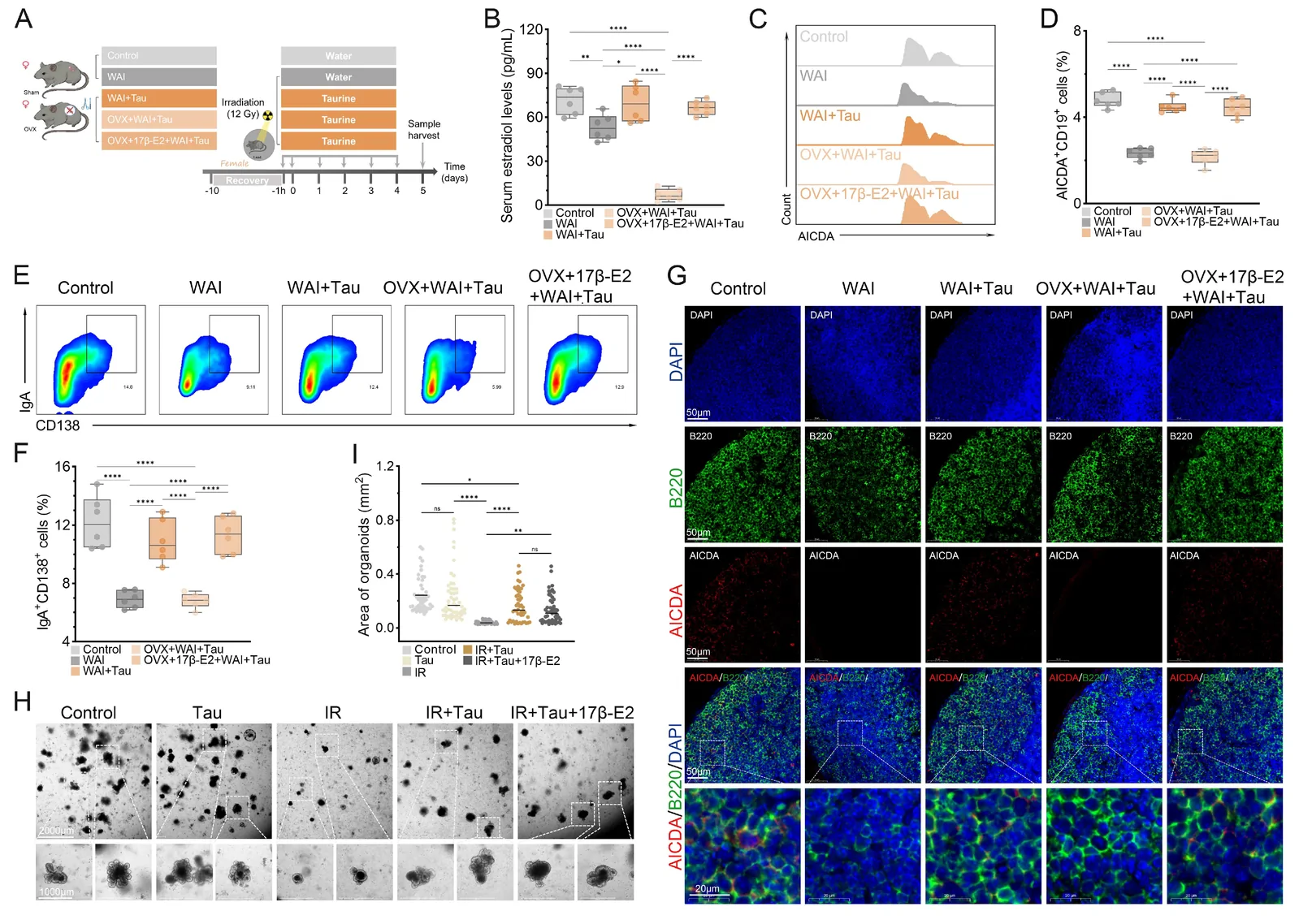
** Estrogen is indispensable for Tau-mediated mucosal immunoprotection *in vivo.*** (A) Schematic diagram of the experimental schematic in OVX mice subjected to WAI. (B) Quantification of serum estradiol levels in the indicated groups. (C, D) Representative flow cytometry plots and quantification (D) of the percentages of AICDA^+^ cells within the CD19^+^ B cell population in the PPs. (E, F) Representative flow cytometry plots (E) and quantification (F) of IgA^+^CD138^+^ plasma cells. (G) Representative immunofluorescence images of lymphoid tissues stained for AICDA (red), B220 (green), and DAPI (blue). Scale bar = 50 μm (upper), 20 μm (lower). (H) Representative bright-field images of in vitro small intestinal organoid formation. Scale bars: 200 μm (upper panels) and 100 μm (lower panels). Data are presented as mean ± SD. (I) Quantification of organoid area. Individual measurements are shown as scatter dots; error bars represent 95% confidence intervals (n = 50). ^*^p < 0.05, ^**^p < 0.01, ^***^p < 0.001, ^****^p < 0.0001, compared with the indicated groups.

**Figure 7:**
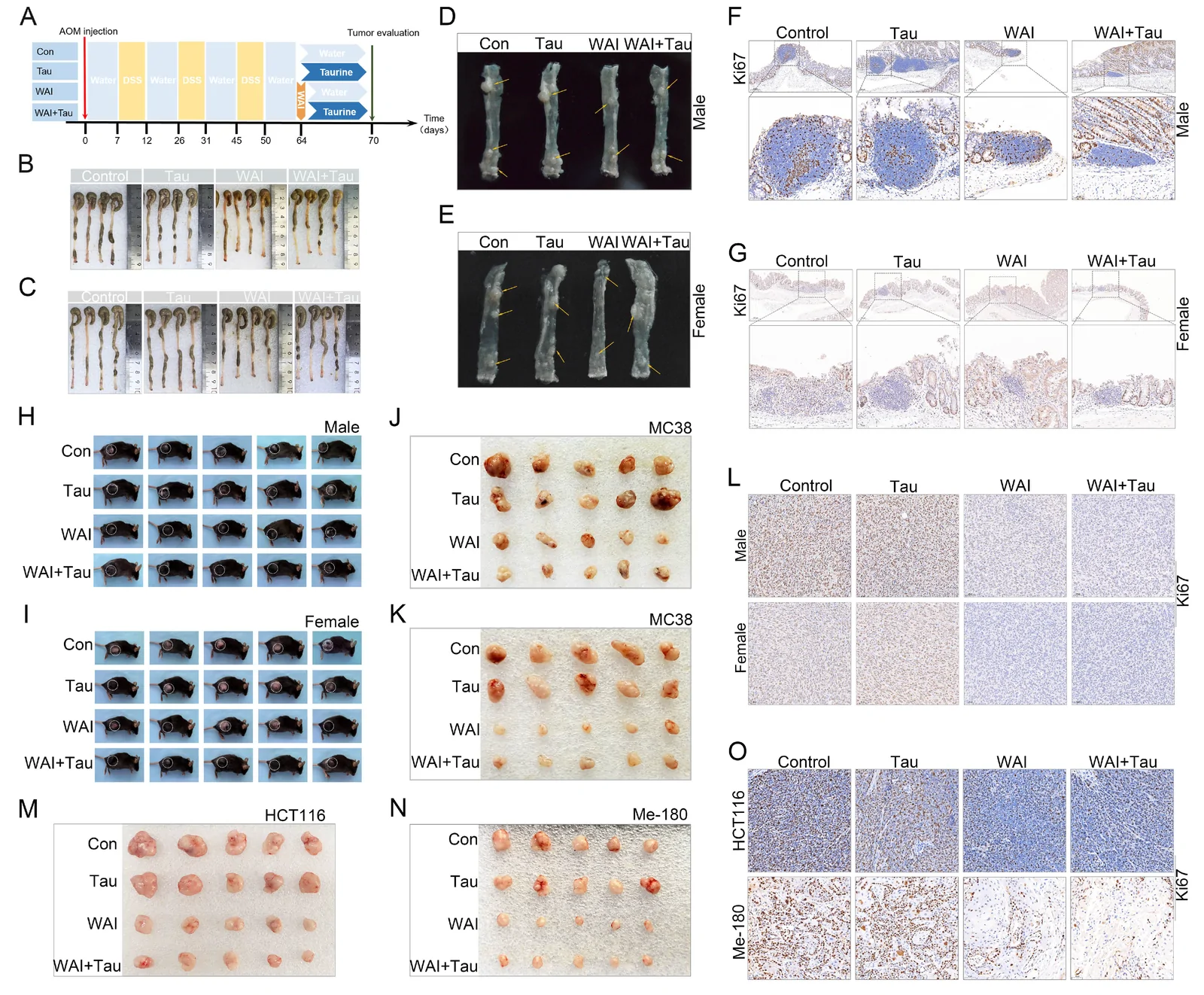
** Tau protects normal tissues against IR without affecting tumor growth or radiosensitivity.** (A) Experimental schematic of AOM/DSS-induced primary colorectal cancer models. (B, C) Representative macroscopic images of intact colons from male (B) and female (C) mice. (D, E) Representative images of longitudinally opened colons showing tumor burden in male (D) and female (E) mice. (F, G) IHC staining of Ki67 in colonic tumor sections from male (F) and female (G) mice. (H, I) Photographs of male (H) and female (I) mice bearing subcutaneous MC38 tumors. (J, K) Excised MC38 tumors from male (J) and female (K) mice after focal radiotherapy and Tau treatment. (L) Representative Ki67 IHC images of MC38 tumor tissues from male and female mice. (M, N) Excised HCT116 (M) and Me-180 (N) xenograft tumors from nude mice following localized irradiation and Tau treatment. (O) IHC evaluation of Ki67 expression in HCT116 and Me-180 tumor tissues.

## Data Availability

The data that support the findings of this study are available from the corresponding author upon reasonable request.

## Abbreviations

AOM: azoxymethane
DSS: dextran sulfate sodium
GO: gene ontology
GSEA: gene set enrichment analysis
H&E: hematoxylin and eosin
IHC: immunohistochemistry
IR: ionizing radiation
ISCs: intestinal stem cells
KEGG: Kyoto Encyclopedia of Genes and Genomes
LEfSe: linear discriminant analysis effect size
NST: normalized stochasticity ratio
OVX: ovariectomy
PAS: periodic acid-Schiff
PCA: principal component analysis
PCoA: principal coordinates analysis
PPs: Peyer's patches
RIII: radiation-induced intestinal injury
Tau: taurine
WAI: whole-abdominal irradiation
