# Supplementary material for: Sex-dependent protective responses of taurine in ameliorating radiation-induced intestinal injury: gut microbiota reprogramming in males and estrogen-dependent immunity in females
Source: Int J Biol Sci. 2026 Aug 24;22(14):7628–43. doi: 10.7150/ijbs.137118 (PMC13617995; doi:10.7150/ijbs.137118)
Supplement: Supplementary file 1 [file ijbsv22p7628s1.pdf]

Supplementary Information

Sex-dependent protective responses of taurine in ameliorating radiation-induced intestinal injury: gut microbiota reprogramming in males and estrogen-dependent immunity in females

Xudong Ding[1], Jia Du[1], Zhaoyu Wang[1], Lu Lu[1, *], Saijun Fan[1, *]

[1] Institute of Radiation Medicine, Chinese Academy of Medical Sciences and Peking Union Medical College, Tianjin Key Laboratory of Radiation Medicine and Molecular Nuclear Medicine, Tianjin 300192, China

***Corresponding authors:**

Lu Lu, lulu@irm-cams.ac.cn

Saijun Fan, fansaijun@irm-cams.ac.cn

Supplementary figures and figure legends

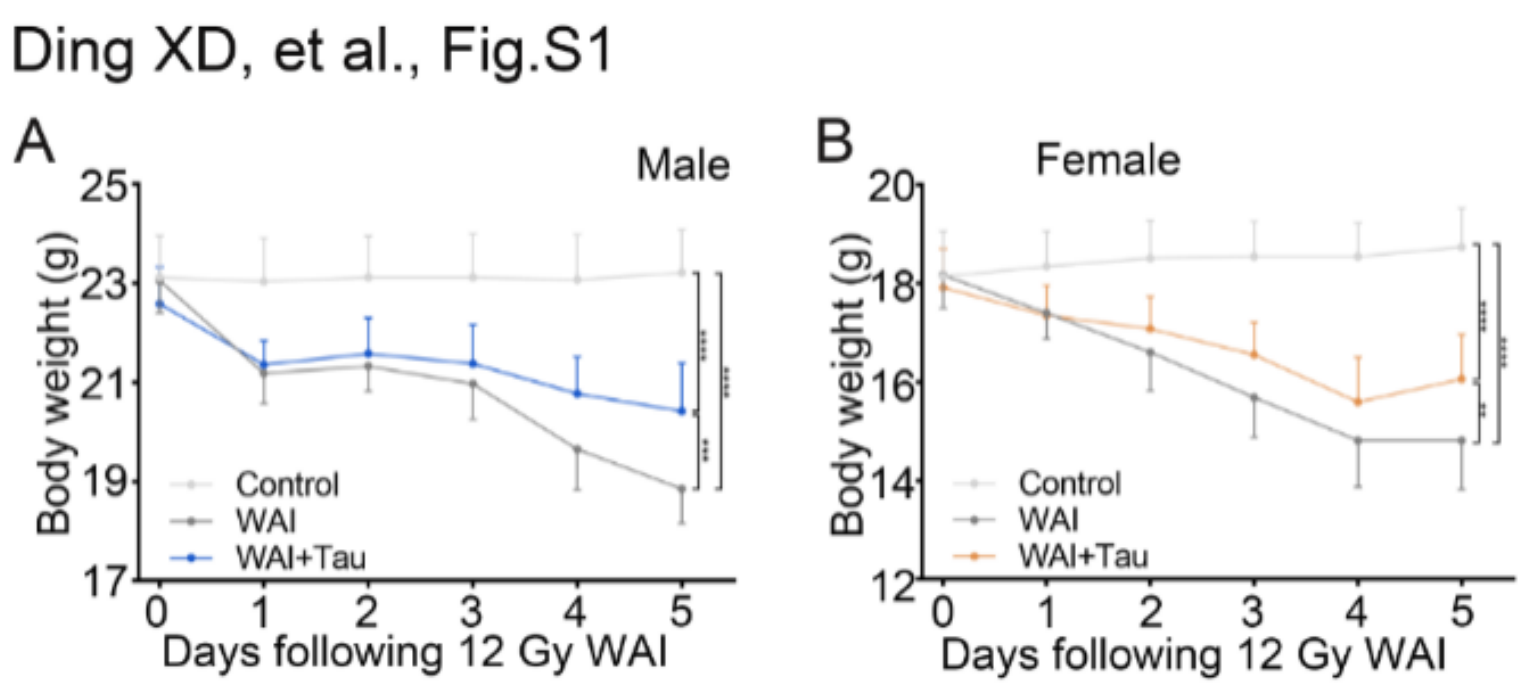

Fig. S1. Daily body weight changes of mice following WAI.

(A, B) Average body weight curves of male (A) and female (B) mice (n=12). Data are presented as mean ± SD. $^{**}p < 0.01$, $^{***}p < 0.001$, $^{****}p < 0.0001$, compared with the indicated groups.

Ding XD, et al., Fig.S2

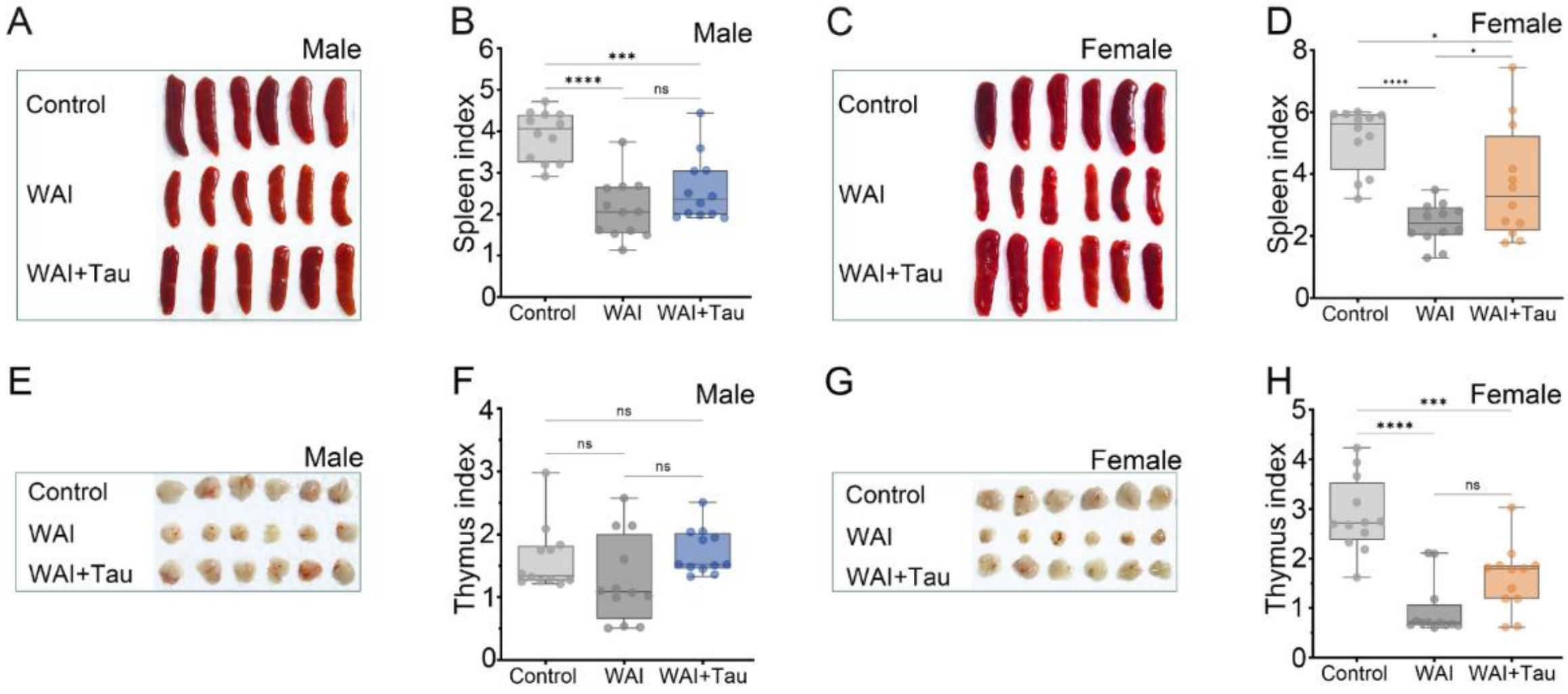

Fig. S2. Tau mitigates WAI-induced splenic and thymic atrophy.

(A, B) Representative macroscopic images of spleens (A) and quantification of the splenic index in male mice (B) (n=12). (C, D) Representative macroscopic images of spleens (C) and quantification of the splenic index in female (D) mice (n=12). (E, F) Representative macroscopic images of thymuses (E) and quantification of the thymic index in male mice (F) (n=12). (G, H) Representative macroscopic images of thymuses (G) and quantification of the thymic index in female mice (H) (n=12). Data are presented as mean ± SD. $^{*}p < 0.05$, $^{***}p < 0.001$, $^{****}p < 0.0001$, compared with the indicated groups.

Ding XD, et al., Fig.S3

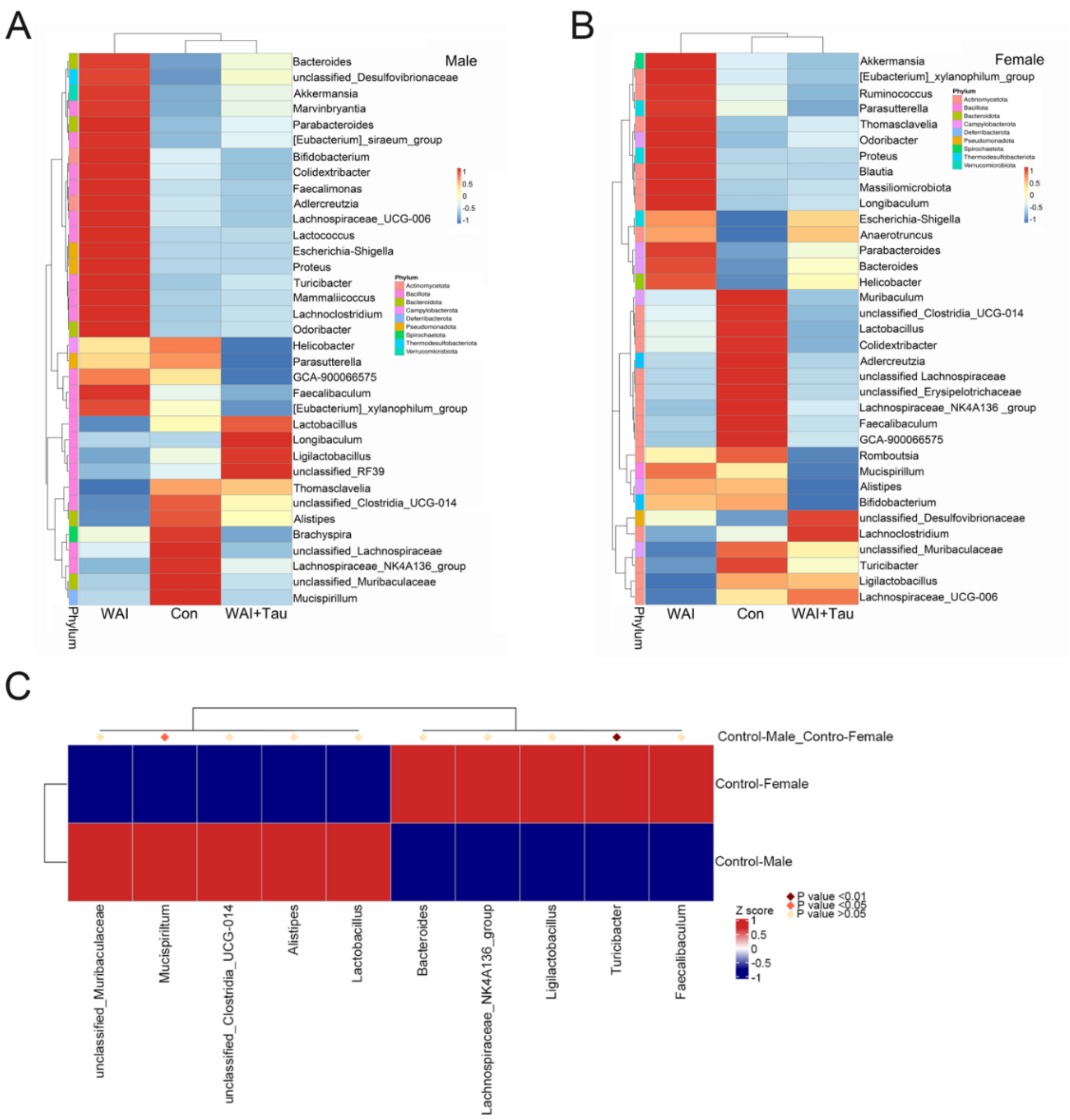

Fig. S3. Tau ameliorates IR-induced gut microbiota dysbiosis.

(A, B) Genus-level clustering heatmaps of the gut microbiota in male (A) and female (B) mice. The colors in the heatmap are based on column Z-scores. The mice with the highest and lowest bacterial levels are denoted by red and blue, respectively. (C) Relative abundance of intestinal flora at the genus level of the gut microbiota in male and female mice.

Ding XD, et al., Fig.S4

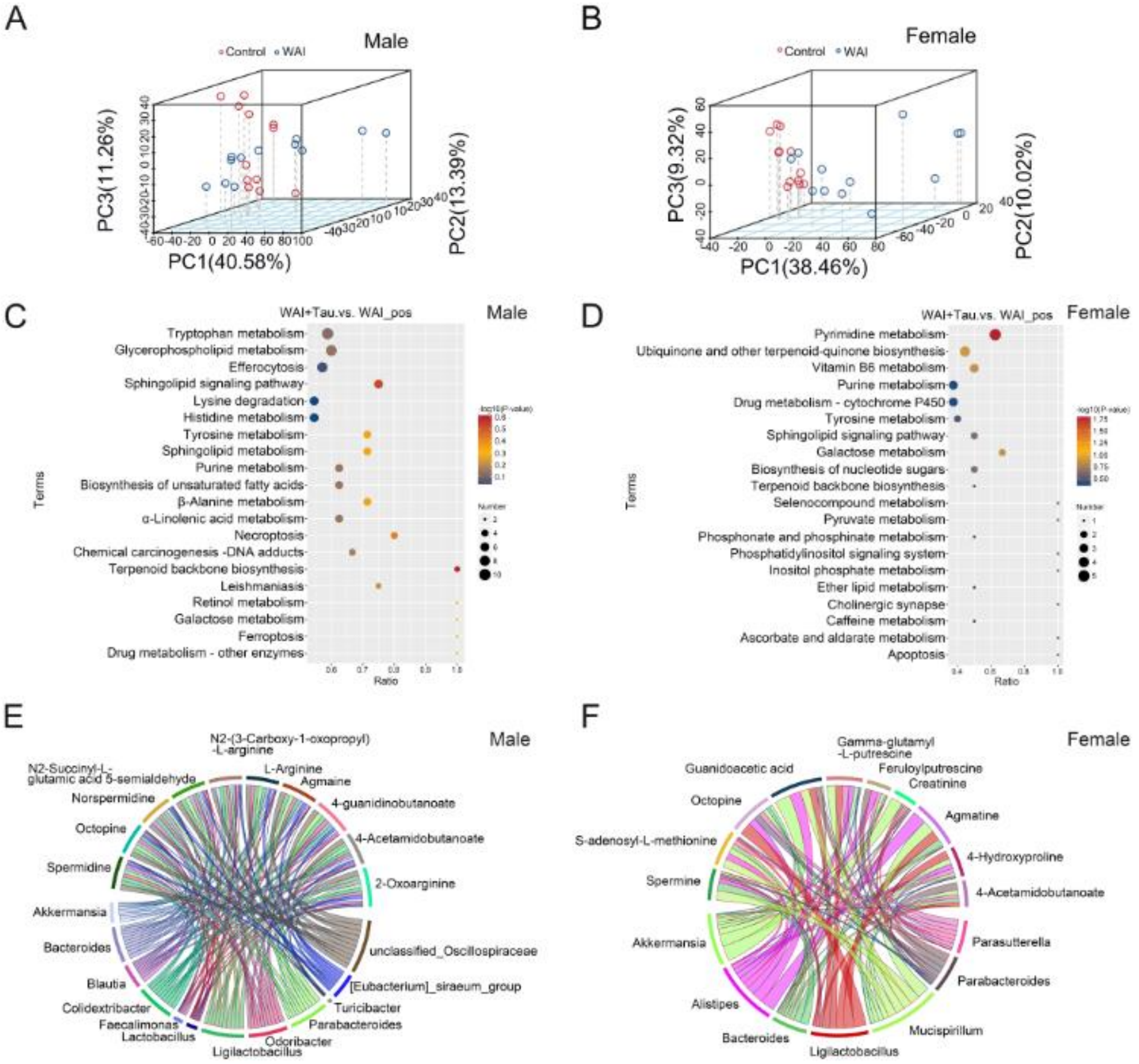

Fig. S4. Irradiation-induced metabolomic alterations and microbiome-metabolome network modulated by Tau.

(A, B) 3D-PCA plots of metabolomic profiles distinguishing the control and WAI groups in male (A) and female (B) mice. (C, D) KEGG pathway enrichment bubble charts of differential metabolites between the WAI+Tau and WAI groups in male (C) and female (D) mice. (E, F) Chord diagrams showing correlations between Tau- modulated gut microbiota and arginine and proline metabolites in irradiated male (E) and female (F) mice.

Ding XD, et al., Fig.S5

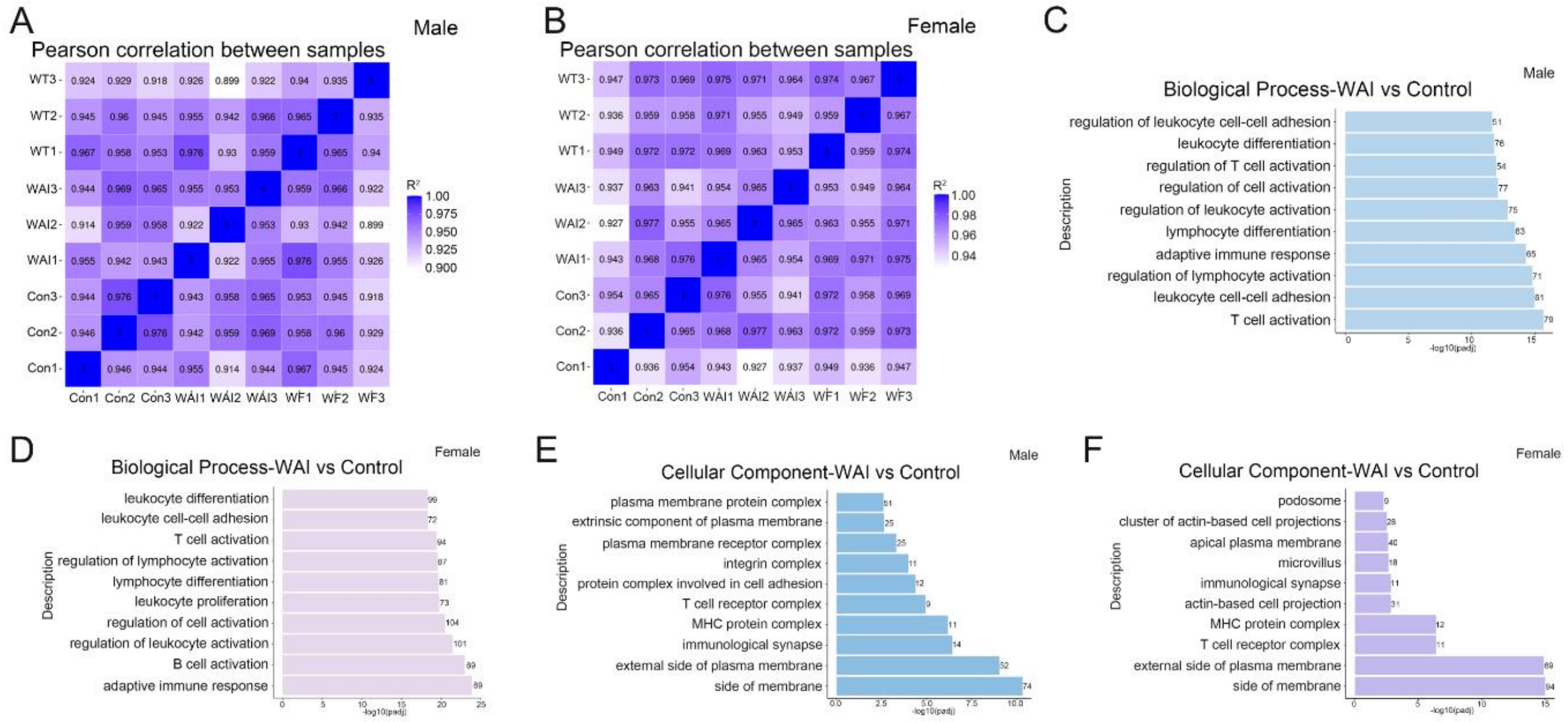

Fig. S5. Transcriptomic sample correlations and supplemental GO enrichment analyses.

(A, B) Pearson correlation heatmaps demonstrating intra-group consistency among male (A) and female (B) samples. (C, D) Biological Process GO enrichment of DEGs between WAI and control groups in male (C) and female (D) mice. (E, F) Cellular Component GO enrichment of DEGs between WAI and control groups in male (E) and female (F) mice.

Ding XD, et al., Fig.S6

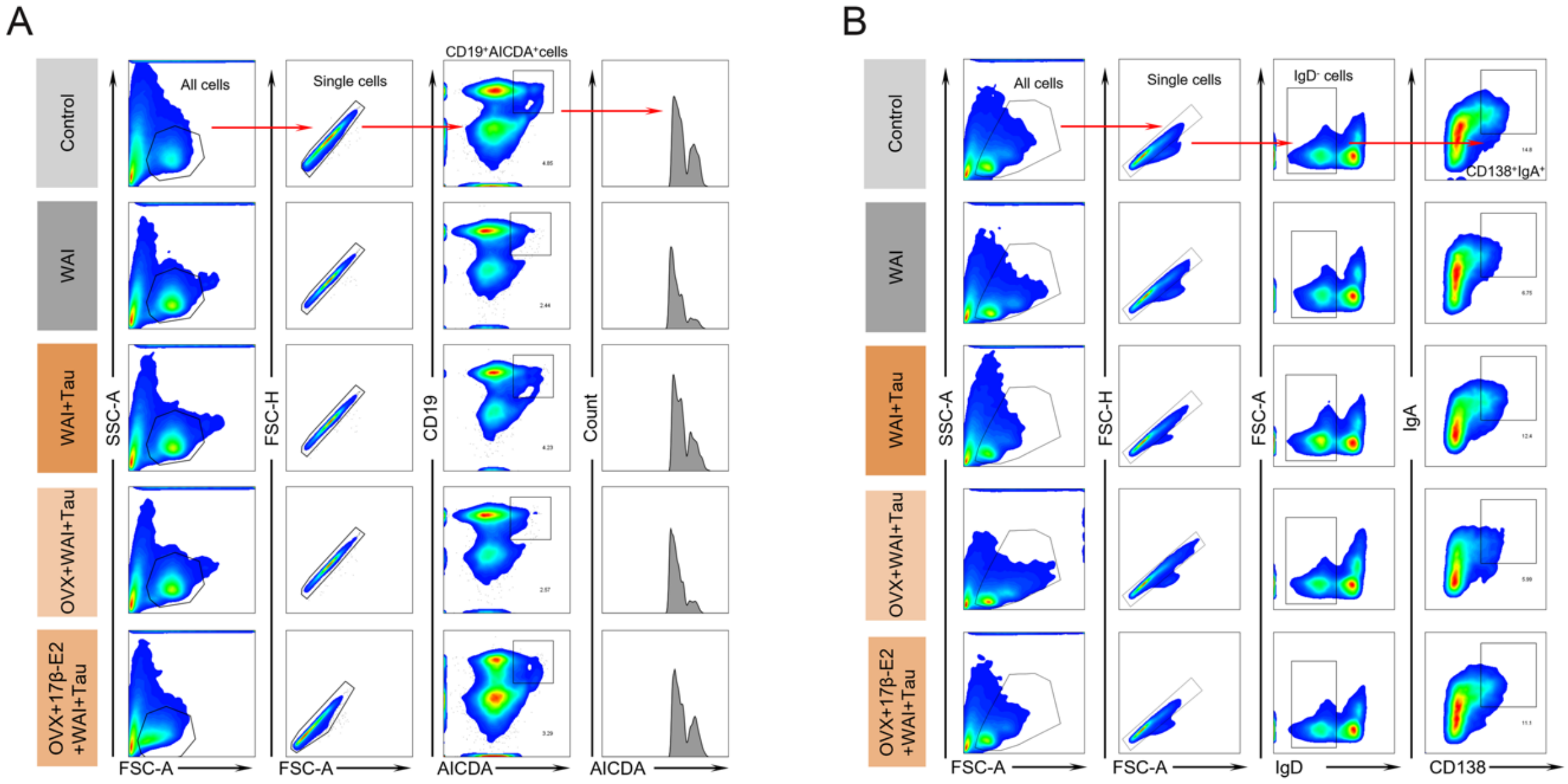

Fig. S6. Flow cytometry gating strategies for evaluating AICDA and IgA upregulation by Tau.

(A) Gating logic and representative plots for assessing AICDA expression within the $CD19^{+}$ B cell population.

(B) Gating strategy identifying immunoglobulin class switching from IgD to IgA, mapping the $CD138^{+}IgA^{+}$ plasma cell subset.

Ding XD, et al., Fig.S7

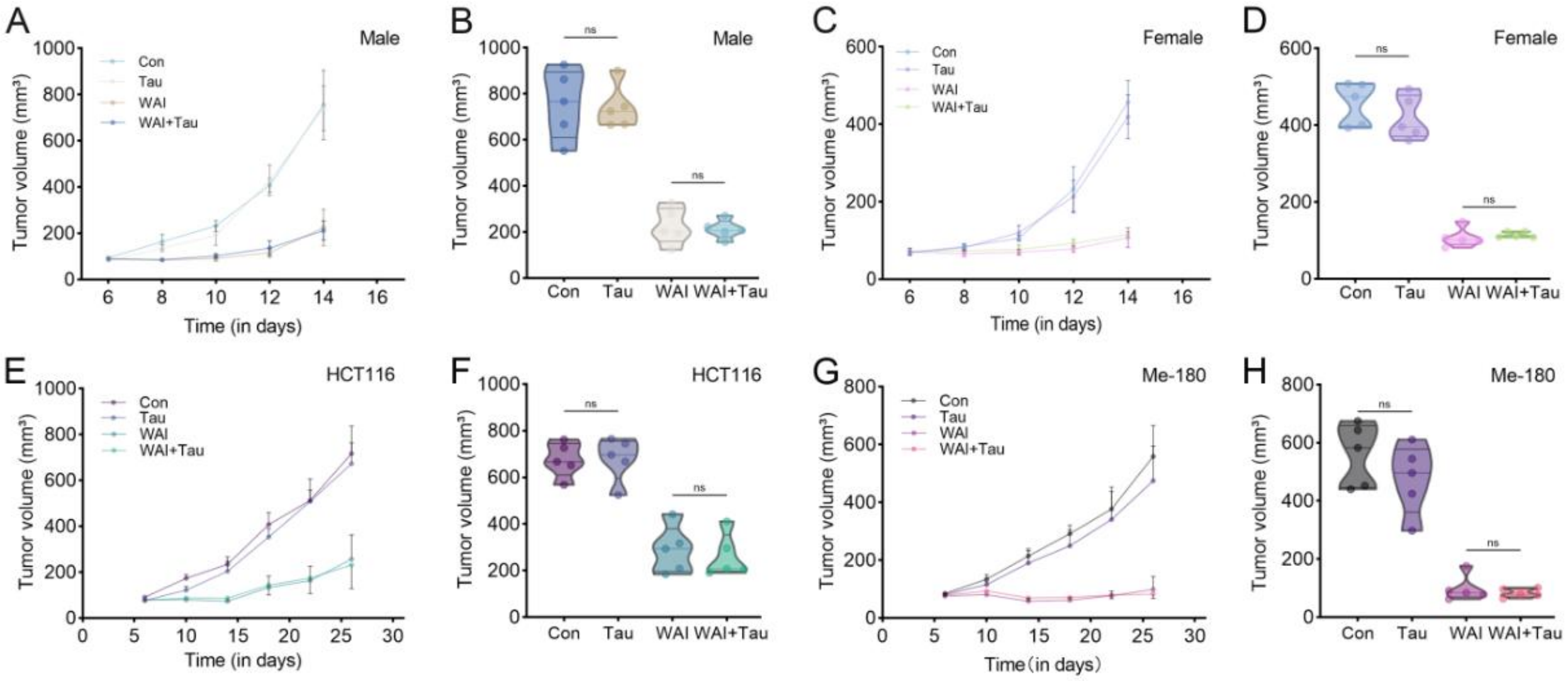

Fig. S7. Effect of Tau on tumor proliferation and radiosensitivity evaluated by growth trajectory analysis.

(A, C) Tumor volume growth curves of MC38 xenografts in male (A) and female (C) C57BL/6J mice. (B, D) Tumor volumes in male (B) and female (D) C57BL/6J mice (n=5). (E, G) Tumor volume growth curves of HCT116 (E) and Me-180 (G) subcutaneous xenografts in nude mice. (F) Tumor volumes of HCT116 xenografts in male nude mice (n=5). (H) Tumor volumes of Me-180 xenografts in female nude mice (n=5). Data are presented as means ± SD.
